# Mitochondrial Probe Methyltriphenylphosphonium (TPMP) Inhibits the Krebs Cycle Enzyme 2-Oxoglutarate Dehydrogenase

**DOI:** 10.1371/journal.pone.0161413

**Published:** 2016-08-18

**Authors:** Moustafa Elkalaf, Petr Tůma, Martin Weiszenstein, Jan Polák, Jan Trnka

**Affiliations:** 1 Laboratory for Metabolism and Bioenergetics, Third Faculty of Medicine, Charles University, Prague, Czech Republic; 2 Department of Biochemistry, Cell and Molecular Biology, Third Faculty of Medicine, Charles University, Prague, Czech Republic; 3 Department of Sport Medicine, Third Faculty of Medicine, Charles University, Prague, Czech Republic; 4 Centre for Research on Diabetes, Metabolism and Nutrition, Third Faculty of Medicine, Charles University, Prague, Czech Republic; National Institute of Environmental Health Sciences, UNITED STATES

## Abstract

Methyltriphenylphosphonium (TPMP) salts have been widely used to measure the mitochondrial membrane potential and the triphenylphosphonium (TPP^+^) moiety has been attached to many bioactive compounds including antioxidants to target them into mitochondria thanks to their high affinity to accumulate in the mitochondrial matrix. The adverse effects of these compounds on cellular metabolism have been insufficiently studied and are still poorly understood. Micromolar concentrations of TPMP cause a progressive inhibition of cellular respiration in adherent cells without a marked effect on mitochondrial coupling. In permeabilized cells the inhibition was limited to NADH-linked respiration. We found a mixed inhibition of the Krebs cycle enzyme 2-oxoglutarate dehydrogenase complex (OGDHC) with an estimated IC_50_ 3.93 [3.70–4.17] mM, which is pharmacologically plausible since it corresponds to micromolar extracellular concentrations. Increasing the lipophilic character of the used TPP^+^ compound further potentiates the inhibition of OGDHC activity. This effect of TPMP on the Krebs cycle ought to be taken into account when interpreting observations on cells and mitochondria in the presence of TPP^+^ derivatives. Compounds based on or similar to TPP^+^ derivatives may also be used to alter OGDHC activity for experimental or therapeutic purposes.

## Introduction

Triphenylphosphonium (TPP^+^) cations have been widely used in the study of mitochondria [1], particularly as probes in the measurement of the mitochondrial membrane potential (mainly methyltriphenylphosphonium salts, TPMP) in isolated mitochondria respiring on succinate [2–4]. In the past decade a number of TPP^+^ derivatives with significant chemical and biological activities have been prepared and used with diagnostic and therapeutic intentions, such as MitoQ, MitoTEMPOL, MitoE, MitoSOD, MitoSOX, MitoB [5–10].

The work of Ross et al. confirmed that the uptake of TPMP follows the Nernst equation Eq (1) with a resultant Δ*ψ*-dependent accumulation inside mitochondria [11].

$$\Delta \psi =\;\frac{RT}{zF}\;\text{ln}\left(\frac{{\left[TPMP^{+}\right]}_{out}}{{\left[TPMP^{+}\right]}_{in}}\right)$$ (1)

Therefore, the accumulation of a monovalent positively charged species such as TPMP^+^ inside a negatively charged compartment at 37°C is given as:

$${\left[TPMP^{+}\right]}_{in}=\;{\left[TPMP^{+}\right]}_{out}\;\times \;10^{-\Delta \psi /61.5}$$ (2)

This corresponds to an approximate ten-fold higher concentration in the cytoplasm with respect to the extracellular space when the plasma membrane potential (Δ*ψ*_*p*_) is -60 mV and another approximate 100-fold accumulation inside the inner mitochondrial compartment with respect to the cytoplasm when the mitochondrial membrane potential (Δ*ψ*_*m*_) is -120 mV.

**Fig 1:**
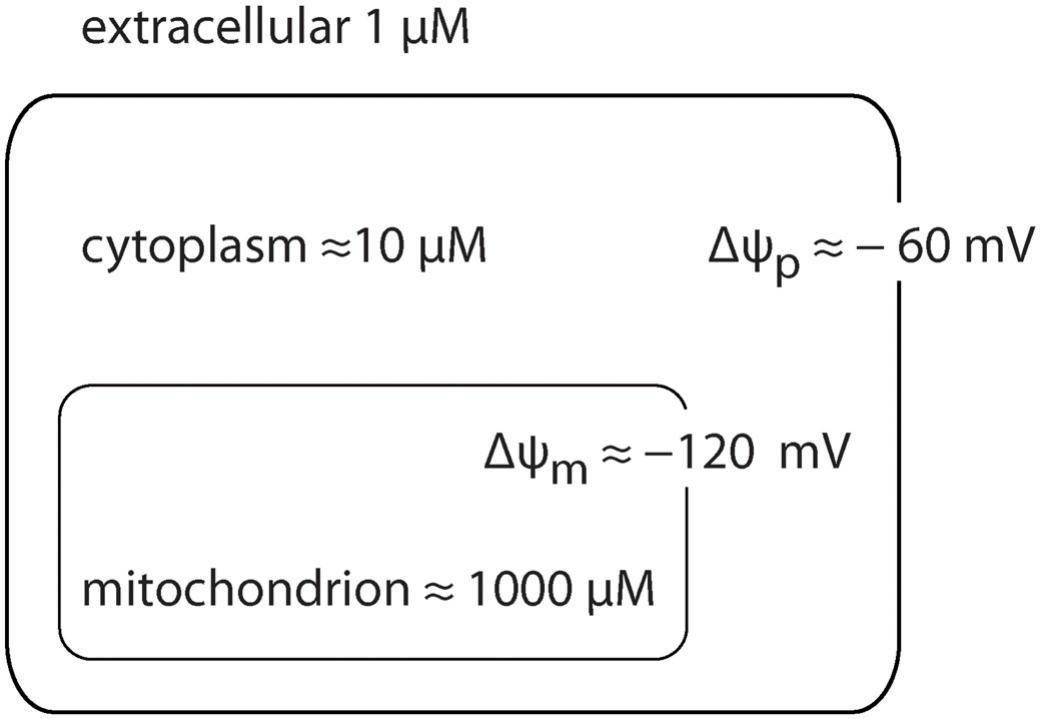
A schematic illustrates TPMP distribution. Due to the membrane potential, lipophilic cations tend to achieve Nernstian equilibrium across biological membranes.

The net result is an approximate 1000-fold accumulation of TPMP inside the mitochondrial matrix compared to the extracellular concentration (Fig 1). Δ*ψ*_*m*_ value varies continuously depending on several factors including the cellular consumption of ATP [12]. The exact concentration of TPMP inside mitochondria hence varies according to changes in Δ*ψ*_*p*_ and Δ*ψ*_*m*_ but it is possible to estimate an approximate concentration in the mitochondrial matrix to be in the millimolar range when the concentration of TPMP in the extracellular space ranges within the micromolar range.

It has been previously observed that some TPP^+^ derivatives exert effects on mitochondrial metabolism [13–15] due to non-specific binding to membranes, such as the potentiation of proton leak across the inner mitochondrial membrane, which is largely independent of the biologically-active moiety but become more pronounced with increasing hydrophobicity of the molecule [16]. The observed inhibitory effect of TPMP on cellular respiration of MES-13 [17] cells indicates that there may be another mechanism of action of TPMP in addition to proton leak. In order to study the effects of TPP^+^ compounds in more detail, we investigated the respiratory response to TPMP in intact cells using the Seahorse XF analyzer [18] and in cells with selectively permeabilized plasma membrane [19] in the presence of various mitochondrial substrates in order to pinpoint, if possible, the specific enzymatic sites of TPMP action. Our results confirmed the previous observation of Reilly et al. [17] and suggested that the inhibitory effect cannot be entirely explained by an interference with mitochondrial membranes or inhibition of respiratory complexes. We therefore investigated the effects of three alkyl-TPP^+^ derivatives on the pyruvate dehydrogenase complex and several other Krebs cycle enzymes and discovered a specific inhibitory effect of TPMP on 2-oxoglutarate dehydrogenase complex (OGDHC). Our study provides a new insight into the inhibitory effect of TPMP and other similar compounds on cellular respiration previously observed in intact cells [17] and in studies with isolated mitochondria [20,21].

## Experimental procedures

### Materials

All chemicals were purchased from Sigma-Aldrich unless stated otherwise.

### Animal care and handling

Wistar rats were obtained from AnLab Ltd., Prague, Czech Republic. The rats were housed in plastic cages (2-4/animals/cage) with free access to food and water and natural light/dark cycle and handled daily to check health status. Animals were euthanized with diethylether overdose prior to tissue isolation. Animal handling and sacrifice took place in a certified animal facility according to principles of laboratory animal care (NIH publication no. 85–23, revised 1985).

### Collection of rat tissues

Three rats 13–15 weeks old weighing 200–300 g were sacrificed. We collected both gastrocnemii muscles to prepare a homogenate enriched in mitochondrial fraction, which was approved for this study by the Committee for Protection of Laboratory Animals of the Third Faculty of Medicine, Charles University in Prague.

### Preparation of muscle homogenate enriched in mitochondrial fraction

We prepared the homogenate by modifying a previously described protocol [22]. A freshly removed rat gastrocnemius was washed three times by ice-cold buffer (250 mM sucrose, 5 mM Tris, 1 mM EGTA, 0.1% fatty acid free BSA, pH 7.4) then flash frozen in liquid nitrogen, and stored at -80°C. On the preparation day, we removed the visible fat and connected tissue by a scalpel blade, and then the muscle was finely dissected into small fragments in a glass dish on ice. The muscle pieces were diluted 1:10 in ice-cold muscle homogenization medium (250 mM sucrose, 20 mM Tris, 40 mM KCl, 2 mM EGTA, pH 7.4) then the suspension was transferred to a glass tube and chopped with an UltraTurrax blender followed by homogenisation in a Dounce homogeniser with a motor-driven Teflon plunger at 500 r.p.m (≈ 10 passes). The homogenate was then centrifuged for 15 min at 600 ×g at 4°C. The supernatant was transferred into new tubes on ice then flash frozen in liquid nitrogen and stored at –80°C. Protein concentration in the homogenate was determined using the BCA assay.

### Cell culture conditions

C2C12 cells were obtained from Sigma-Aldrich and grown in Dulbecco-modified Eagle's medium (DMEM, Life Technologies) containing 1 g/l D-glucose to enhance mitochondrial respiration [23], 4 mM L-glutamine and 1 mM sodium pyruvate, and supplemented with 10% fetal bovine serum, 100 unit/ml penicillin and 100 μg/ml streptomycin. All cultures were incubated at 37°C in a humidified atmosphere containing 5% CO_2_ and 95% air. Cells were subcultured every 48 hours.

### Analysis of metabolism in intact cells

Cellular respiration of intact C2C12 cells was measured using the XF-24 analyzer (Seahorse Bioscience). We performed mitochondrial bioenergetic assays based on published protocols [18]. The XF assay medium (bicarbonate-free modified DMEM, Seahorse Bioscience) was supplemented with 4 mM L-glutamine, 1 mM pyruvate, and 1 g/l D-glucose. The pH was adjusted with 1 M NaOH to 7.4 at 37°C. Cells were seeded at a density of 20,000 cells per well and left overnight to attach and proliferate to obtain a monolayer of cells before measurement. After measuring the basal respiration, TPMP or vehicle (deionized H_2_O) were injected and a mitochondrial respiration test was performed by sequential additions of 1 μM oligomycin, 0.5 μM carbonyl cyanide-4-(trifluoromethoxy)phenylhydrazone (FCCP) and 1 μM rotenone and antimycin A.

### Analysis of metabolism in permeabilized cells

We assayed respiratory activity in permeabilized C2C12 myoblast using the Seahorse XF-24 analyzer by modifying a published protocol [19]. Cells were seeded as previously mentioned. The mitochondrial assay solution (MAS) contained 70 mM sucrose, 220 mM mannitol, 10 mM KH_2_PO_4_, 5 mM MgCl_2_, 2 mM HEPES, 1 mM EGTA, 0.2% BSA, and 4 mM ADP. The pH was adjusted with 10 M KOH to 7.4 at 37°C. The used substrates included 5 mM pyruvate/ 2.5 mM malate, 5 mM glutamate/ 2.5 mM malate or 10 mM succinate and 10 μM rotenone. Cellular permeabilization was performed using the XF plasma membrane permeabilizer reagent (XF-PMPR, Seahorse Biosciences) at a final concentration of 1 nM prior to the measurement. After measuring OCR in basal state, 10 μM TPMP or vehicle (deionized H_2_O) were injected, followed by injection of 0.5 μM FCCP and 1 μM antimycin A.

### Enzymatic activity

The activity of enzymes was assayed spectrophotometrically using TECAN Infinite M200Pro microplate reader. To find the site of inhibition, we assayed all enzymes in a homogenate enriched in mitochondrial fraction prepared from rat skeletal muscle except the pyruvate dehydrogenase complex, which was obtained purified (Sigma P7032) due to high interference of lactate dehydrogenase in the homogenate even after inhibition with sodium oxamate. Prior to enzymatic assays this homogenate was exposed to three cycles of rapid freeze- thaw. In assays where the absorbance of NADH was followed, 10 μM rotenone and 1 μM antimycin A were added to block the activity of respiratory chain complexes. We modified the published protocols mainly to fit the assays in the microplate reader. To study the kinetics of OGDHC and the activity of its components, we used the purified OGDHC enzyme (Sigma K1502).

#### Pyruvate dehydrogenase complex (PDHC)

Using the provider’s protocol, in assay mixture containing 50 mM Tris (pH 7.4), 0.2 mM MgCl_2_, 10 μM CaCl_2_, 2 mM NAD^+^, 2.6 mM L-cysteine, 0.12 mM coenzyme A sodium, 2.5 mM L-carnitine, 0.3 mM thiamine pyrophosphate, 1 mM sodium pyruvate and 0.005 U/ml pyruvate dehydrogenase. A_340_ was followed for 3 min at 30°C.

#### Citrate synthase (CS)

As previously described [22], in assay mixture containing 100 mM Tris (pH 8), 0.1% Triton X-100, 0.1 mM 5,5′-dithiobis(2-nitrobenzoic acid) (DTNB), 0.3 mM acetyl coenzyme A trilithium and 0.5 mM freshly prepared sodium oxaloacetate. A_412_ was followed for 3 min at 30°C.

#### NAD^+^ dependent isocitrate dehydrogenase (IDH)

Using a modified protocol [24], in assay mixture containing 70 mM Tris-acetate (pH 7.6), 0.1% Triton X-100, 0.1 mM EDTA, 10 μM rotenone, 1 μM antimycin A, 2.5 mM MnCl_2_, 0.8 mM NAD^+^, 1 mM ADP, 1 mM DTT and 8 mM trisodium isocitrate. A_340_ was followed for 3 min at 30°C.

#### 2-Oxoglutarate dehydrogenase complex (OGDHC)

Using a modified protocol [25], in assay mixture containing 100 mM Tris (pH 8), 0.1% Triton X-100, 0.1 mM EDTA, 10 μM rotenone, 1 μM antimycin A, 2 mM MgCl_2_, 2 mM CaCl_2_, 0.8 mM NAD^+^, 2 mM DL-Dithiothreitol (DTT), 0.5 mM coenzyme A sodium, 0.1 mM thiamine pyrophosphate and 2 mM 2-oxoglutarate monobasic. A_340_ was followed for 3 min at 30°C.

#### Glutamate dehydrogenase (GDH)

Using a modified protocol [26], in assay mixture containing 100 mM Tris (pH 9.1), 0.1% Triton X-100, 10 μM rotenone, 1 μM antimycin A, 1 mM CaCl_2_, 2 mM CaCl_2_, 0.5 mM NAD^+^ and 10 mM glutamate. A_340_ was followed for 5 min at 30°C.

#### Malate dehydrogenase (MDH)

Using a modified protocol [25], in assay mixture containing 100 mM (pH 8) Tris, 0.1% Triton X-100, 0.1 mM EDTA, 10 μM rotenone, 1 μM antimycin A, 2.5 mM MgCl2, 0.8 mM NAD^+^, 1 mM DTT, 0.3 mM acetyl CoA trilithium and 10 mM sodium malate. Acetyl CoA was added to accelerate the elimination of oxaloacetate to obtain a linear reaction. A_340_ was followed for 3 min at 30°C.

#### Complex I

Using a modified protocol [27], in assay mixture composed of 25 mM potassium phosphate (pH 7.8), 3.5 g/l BSA, 2 mM EDTA, 60 μM 2,6-dichloroindophenol (DCIP), 70 μM decylubiquinone, 1 μM antimycin A and 0.2 mM NADH. A_600_ was followed for 3 min at 37°C. Rotenone sensitive activity was calculated by subtracting the activity of wells treated with 10 μM rotenone.

#### Complex II

Using a modified protocol [27], in assay mixture containing 80 mM potassium phosphate (pH 7.8), 1 g/l BSA, 2 mM EDTA, 10 mM succinate, 80 μM DCIP, 50 μM decylubiquinone, 1 μM antimycin A and 3 μM rotenone. A_600_ was followed for 3 min at 37°C. Malonate sensitive activity was calculated by subtracting the activity of wells treated with 20 mM malonate.

#### Complex III

According to [28], in assay mixture containing 25 mM potassium phosphate (pH 7.5), 50 μM ferricytochrome *c*, 4 mM sodium azide, 0.1 mM EDTA, 0.025% Tween^®^ 20 and 50 μM decylubiquinol. A_550_ was followed for 3 min at 37°C. Antimycin A sensitive activity was calculated by subtracting the activity of wells treated with 10 μM antimycin A. Decylubiquinol was prepared by dissolving decylubiquinone in acidified ethanol (pH 4), then few grains of sodium borohydride (NaBH_4_) were added followed by vortexing until the solution became colorless [29].

#### Complex IV

As previously described [30], in assay buffer containing 30 mM potassium phosphate (pH 7.4) and 25 μM of freshly prepared ferrocytochrome *c*. A_550_ was followed for 3 min at 37°C. The absorbance of samples oxidised with 10 μl of 0.5 M potassium hexacyanoferrate(III) was subtracted from all measurements, then the natural logarithm of absorbance was plotted against time and compared to untreated control. Ferrocytochrome *c* was freshly prepared by adding few grains of sodium dithionite to 1 mM stock of ferricytochrome *c*.

#### Purified OGDHC enzyme studies

We used purified OGDHC to study the effect of TPMP on OGDHC kinetics using Michaelis-Menten model and the impact on each component of the complex. We incubated purified OGDHC in reaction mixture containing different concentrations of TPMP (0, 0.5, 1, 1.5, 2, 3, 4, 5 mM) and induced the reaction by adding different concentrations of 2-oxoglutarate (0, 0.05, 0.1, 0.25, 0.5, 1 mM). We concluded the values of Vmax and Km by non-linear curve fit and then plotted these values against TPMP concentrations to visualize the relationship between Vmax or Km and TPMP concentration. We followed that with a linear regression analysis.

The activity of OGDHC was measured according to the provider’s protocol in assay mixture containing 50 mM Tris (pH 7.4), 0.2 mM MgCl_2_, 10 μM CaCl_2_, 2 mM NAD^+^, 2.6 mM l-cysteine, 0.12 mM coenzyme A sodium, 0.3 mM thiamine pyrophosphate, 0.005 U/ml OGDHC and sodium 2-oxoglutarate monobasic. A_340_ was followed for 3 min at 30°C. We assayed the enzymatic activity of individual components of OGDHC as previously described [31]. E1 component (alpha-ketoglutarate dehydrogenase) was assayed in 35 mM KH_2_PO_4_ (pH 7.5), 0.5 mM EDTA, 0.5 mM MgSO_4_, 0.1 mM DCIP, 1 mM thiamine pyrophosphate, 0.1 U/ml OGDHC and 2 mM sodium 2-oxoglutarate monobasic. A_600_ was followed for 3 min at 30°C. E2 component (dihydrolipoyl transsuccinylase) was assayed in 35 mM KH_2_PO_4_ (pH 7.5), 0.5 mM EDTA, 0.1 mM DTNB, 0.1 U/ml OGDHC and 0.1 mM succinyl coenzyme A sodium. A_412_ was followed for 3 min at 30°C. E3 component (dihydrolipoyl dehydrogenase) was assayed in 35 mM KH_2_PO_4_ (pH 7.5), 0.5 mM EDTA, 0.3 NADH, 0.1 U/ml OGDHC and 0.4 mM lipoamide. A_340_ was followed for 3 min at 30°C.

### Metabolite level determination

Determination of low molecular organic acids was performed by a capillary electrophoretic procedure combined with a highly sensitive contactless conductivity detection as previously described [32,33]. The cells were trypsinized then centrifuged and resuspended in serum free medium, then treated with 10 μM TPMP or vehicle deionized H_2_O) in suspension (≈ 4 million cells) with continuous gentle shaking for one hour in a water bath at 37°C. Cells were then centrifuged and samples from the supernatant were obtained for analysis of extra-cellular lactate. The rest of supernatant was discarded and the pellet was extracted to 100 μl 75% acetonitrile/water *v/v* and vigorously vortexed. The mixture was centrifuged at 5000 ×g at 4°C, then the supernatant was carefully taken to measure citric acid and 2-oxoglutaric acid. All electrophoretic measurements were carried out using the HP^3D^CE system (Agilent Technologies, Waldbronn Germany) in an uncoated fused silica capillary (Composite Metal Services, UK), inner diameter 25 μm, outer diameter 360 μm, total length 31.5 cm, length to detector 18 cm. 2-Oxoglutaric acid was determined in the optimized background electrolyte of 50 mM citric acid + 2% *m/v* polyethylene glycol (PEG, M_r_ 8000) dissolved in 50% *v/v* acetonitrile, pH 2.5; at a separation voltage/current -30 kV/-2.5 μA. Electrophoretic separation of citric and lactic acids was carried out in the background electrolyte of 20 mM N-cyclohexyl-2-aminoethanesulfonic acid (CHES)/NaOH, pH 9.5; at a separation voltage/current +30 kV/+6 μA.

### Detection of changes in mitochondrial membrane potential Δψ_m_

Qualitative changes in Δ*ψ*_*m*_ were determined as the changes in tetramethylrhodamine methyl ester (TMRM) fluorescence in C2C12 myoblasts [34,35]. Cells were seeded in a 96 well plate (≈ 30,000 cells/well) and left to attach over night. After careful washing and to perform the measurement in the non-quench mode [36], cells were incubated with 10 nM TMRM and the relevant treatment for 45 minutes at 37°C in serum-free, phenol red-free DMEM (Life technologies) supplemented with 4 mM L-glutamine, then washed with medium containing 0.2% fatty acid free BSA, and incubated again for 5 minutes before bottom TMRM fluorescence was measured with TECAN Infinite M200Pro microplate reader (ex 520 ±9 nM, em 580 ±20 nM) at 37°C.

### Statistical analyses

Data are presented as mean [95% confidence interval (CI)]. Student’s t-test with alpha level set to 0.05 or one-way ANOVA with Tukey's multiple comparisons test were performed using GraphPad Prism version 6.0d for Mac OS X, GraphPad Software, La Jolla California USA, www.graphpad.com. We used the same program for the regression analysis and curve fitting to estimate IC_50_, Vmax and Km values. Differences found statistically significant are marked with an asterisk. The number of independent experiments is denoted as n.

## Results

### TPMP inhibits mitochondrial respiration in intact cells

**Fig 2:**
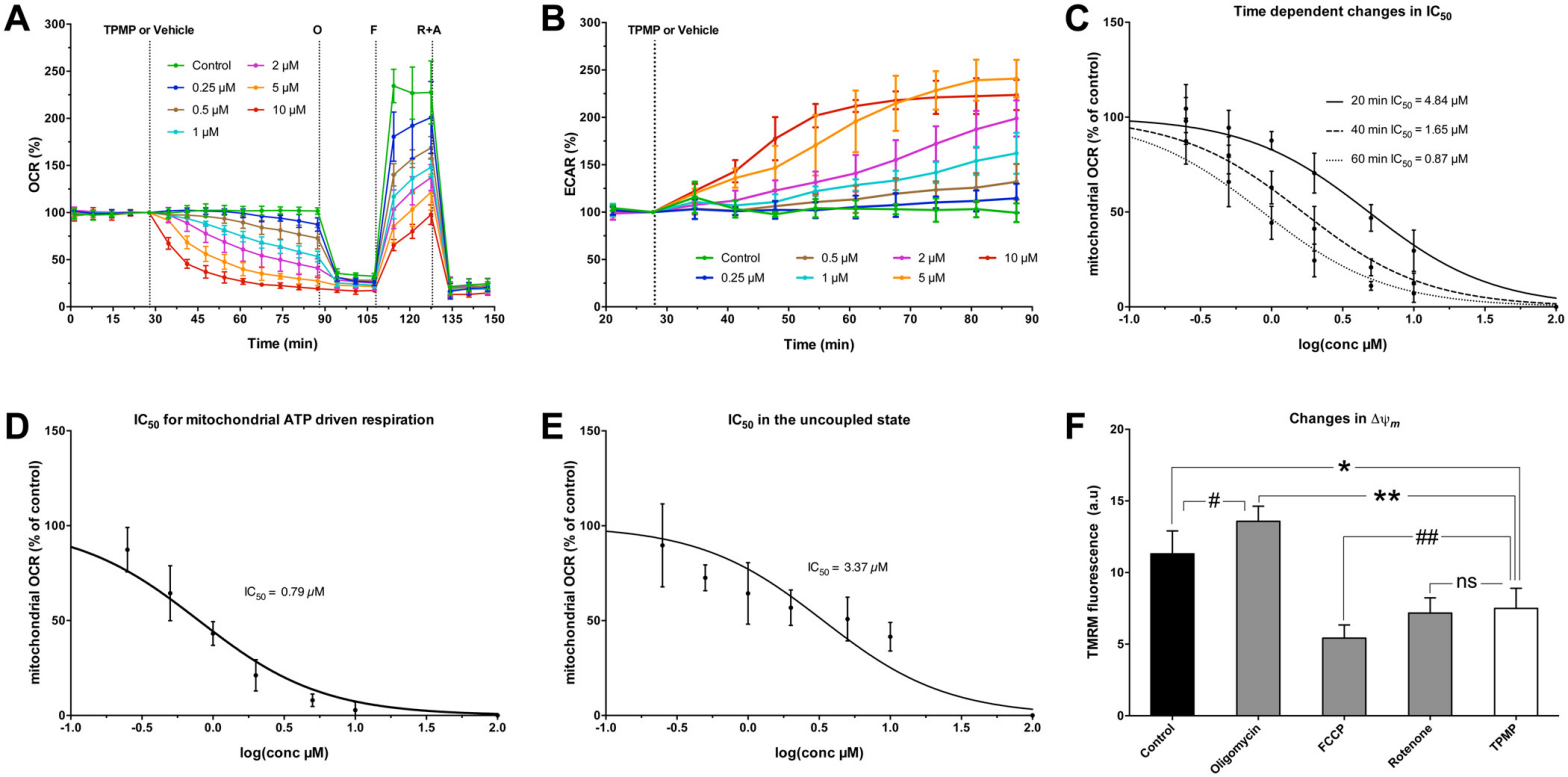
TPMP inhibits mitochondrial respiration and increases glycolytic activity in intact C2C12 cells. **A.** Metabolic analysis of cellular respiration in intact C2C12 myoblasts. Cells were treated with different TPMP concentrations or vehicle (deionized H_2_O), followed by a sequential injection of 1 μM oligomycin (O), 1 μM FCCP (F), then 1 μM rotenone and antimycin A (R+A) mixture. The TPMP treated cells showed a decrease in oxygen consumption rate (OCR) following the TPMP addition. The inhibitory effect following TPMP treatment is demonstrated by a gradual dose-dependent decrease in cellular OCR. Data is expressed as the percentage of basal OCR (OCR%) and is presented as means ±95% CI, n = 4, measured in triplicate. **B.** A corresponding increase in ECAR following TPMP addition. Data is expressed as the percentage of basal ECAR (ECAR%) and is presented as means ±95% CI, n = 4, measured in triplicate. **C.** IC_50_ shift showing the time dependent inhibition after 20, 40, and 60 minutes of TPMP treatment. **D.** IC_50_ for mitochondrial ATP driven respiration represents difference between respiration after oligomycin addition and respiration in the steady state, which includes proton leak respiration. **E.** An increase in IC_50_ of uncoupled respiration following the induced collapse of membrane potential by FCCP and the leak of the accumulated TPMP inside mitochondria. In C, D & E mitochondrial OCR was calculated by subtracting non-mitochondrial respiration after rotenone-antimycin A treatment, then the values were plotted against logarithm values of TPMP in micromolar concentration (log TPMP μM vs. normalized response %). IC_50_ was then estimated by non-linear regression analysis. **F.** Detection of changes in Δ*ψ* induced by 10 μM TPMP and mitochondrial inhibitors expressed as changes in TMRM fluorescence (ex = 520 nm and em = 580 nm). We perturbed mitochondrial respiration by different inhibitors to induce different states of membrane polarization. The groups included an untreated control (polarized), 1 μM oligomycin treated (hyperpolarized), 0.5 μM FCCP treated (depolarized) and 1 μM rotenone treated (decreased polarization). Data is expressed as TMRM fluorescence (a.u) means ±95% CI, n = 4, in which each group included 10 wells. *, p = 0.0013; #, p = 0.0094; **, p<0.0001; ##, p = 0.0076; ns, not significant.

An acute exposure to TPMP caused a gradual decrease in oxygen consumption rate in intact C2C12 cells respiring on glucose, glutamine and pyruvate in a dose-dependent manner (Fig 2A). The corresponding increase in the extracellular acidification rate was indicative of anaerobic glycolysis (Fig 2B). The extent of the decrease in respiration was time-dependent, which was demonstrated by the time-dependent shift in the IC_50_ from 4.84 μM after 20 minutes to reach 0.87 μM after 60 minutes (Fig 2C). TPMP inhibited mainly mitochondrial ATP synthesis-driven respiration (S1 Fig), the IC_50_ for this parameter was found to be 0.79 μM (Fig 2D). Removing the regulatory function of ATP synthase by uncoupling with FCCP caused a rise in the IC_50_ to be 3.37 μM (extracellular) presumably due to the leak of TPMP from mitochondria following the collapse of membrane potential (Fig 2E).

The inhibitory effect on respiration makes it difficult to assess TPMP-induced proton leak by an analysis for respiration alone. Therefore we decided to measure mitochondrial membrane potential using a membrane-permeable cationic fluorophore. When we compared the effect of TPMP with other mitochondrial inhibitors on Δ*ψ*_*m*_ we found that TPMP caused a decrease in Δ*ψ*_*m*_ but not as much as the protonophore FCCP that causes complete collapse of membrane potential. The ATP synthase inhibitor oligomycin induced a hyperpolarization, as expected. The complex I inhibitor rotenone (Fig 2F) caused a similar decrease in membrane potential as TPMP, in this case by decreasing the influx of electrons into the respiratory chain, which suggests a similar action by TPMP.

### Inhibition of NADH-linked respiration in permeabilized cells

**Fig 3:**
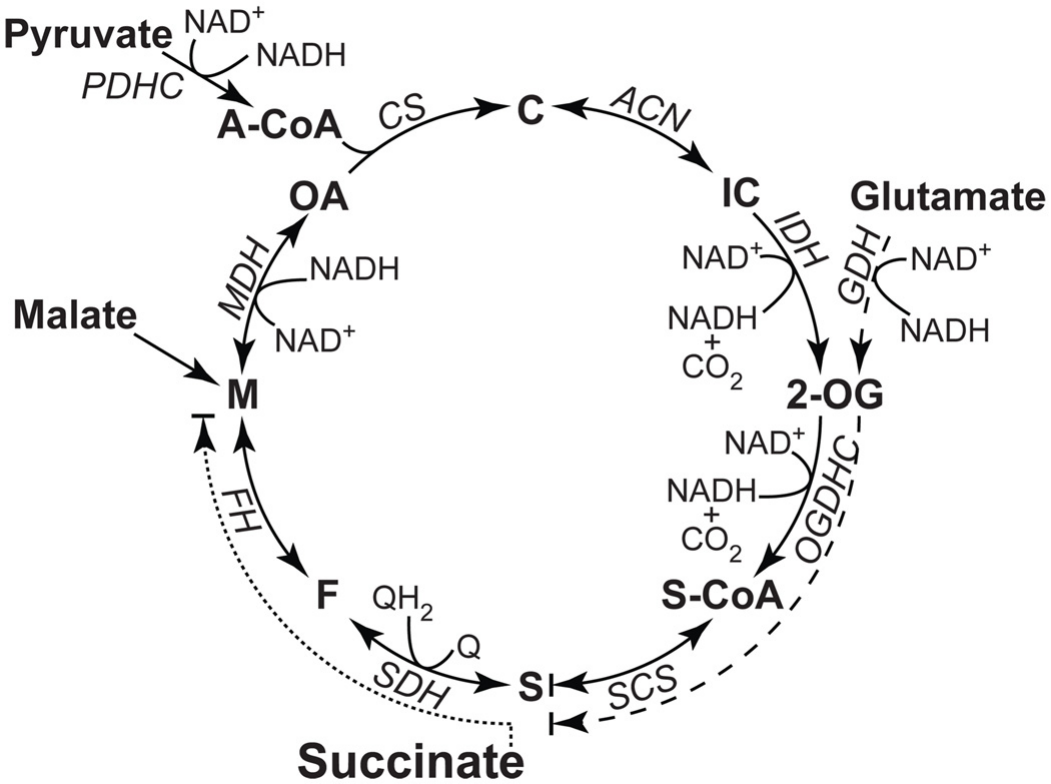
A schematic presentation of Krebs cycle showing the active enzymes when different mitochondrial substrates are used. NADH produced is re-oxidized by the respiratory complex I. The role of malate in pyr/mal respiration is to provide oxaloacetate that is further metabolized to citrate in the presence of acetyl-CoA. In glu/mal respiration, malate takes part in the malate aspartate shuttle. PDHC, pyruvate dehydrogenase complex; A-CoA, acetyl-CoA; CS, citrate synthase; C, citrate; ACN, aconitase; IC, isocitrate; IDH, isocitrate dehydrogenase; GDH, glutamate dehydrogenase; 2-OG, 2-oxoglutarate; OGDHC, 2-oxoglutarate dehydrogenase complex; S-CoA, succinyl-CoA; SCS, succinyl-CoA synthase; S, succinate; SDH, succinate dehydrogenase; F, fumarate; FH, fumarate hydratase; M, malate; MDH, malate dehydrogenase; OA, oxaloacetate; Q, ubiquinone; QH_2_, ubiquinol.

We then studied the effects of TPMP in cells with permeabilized plasma membrane, which allowed us to use a wider range of respiratory substrates that fit differently into mitochondrial metabolism, namely pyruvate/malate and glutamate/malate, which feed into the Krebs cycle and via complex I into the respiratory chain, and succinate, which is metabolized solely by the respiratory complex II (Fig 3).

**Fig 4:**
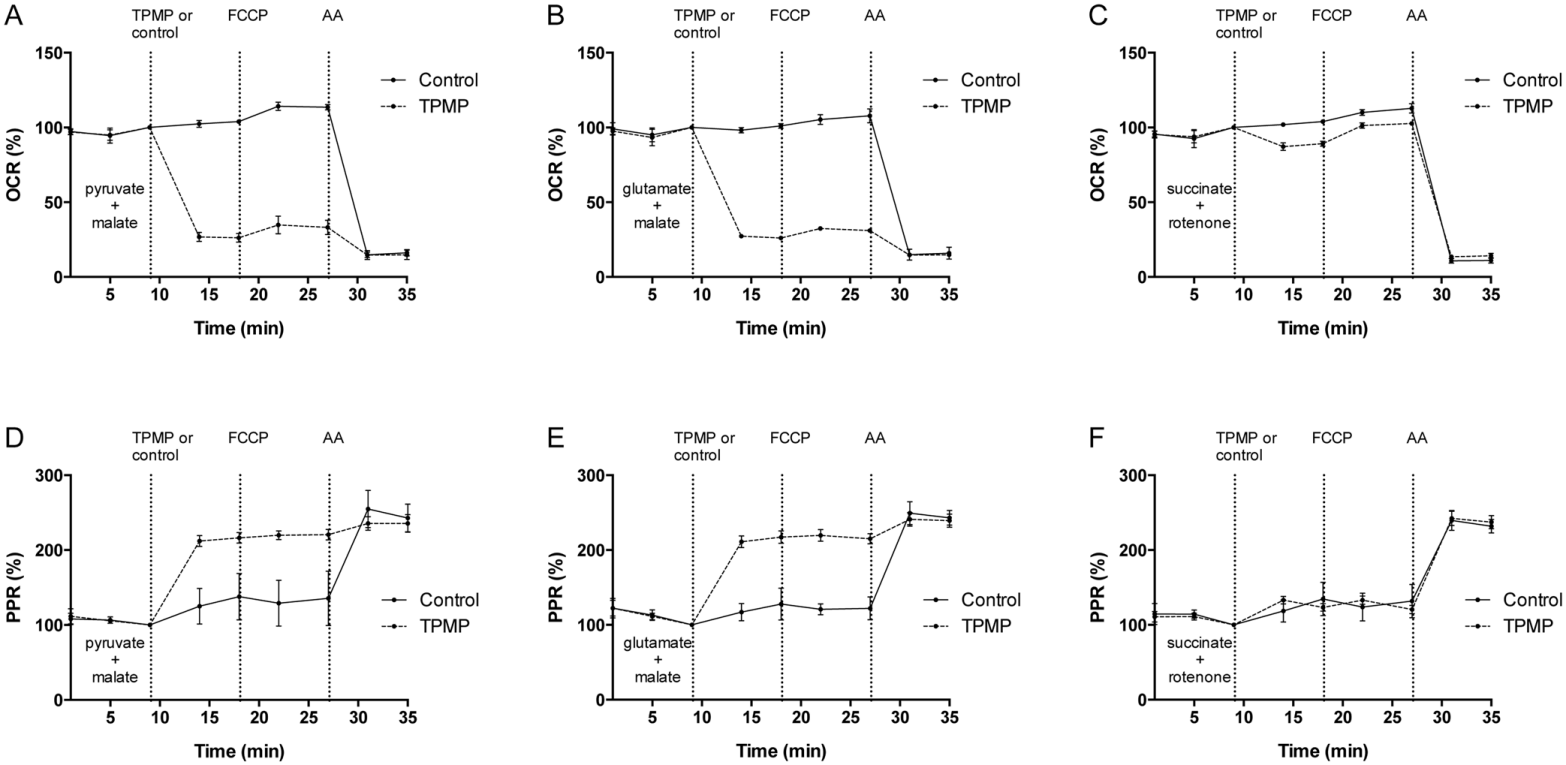
Respiration in permeabilized C2C12 cells in the presence of different metabolic substrates. Respiration was induced by the addition of ADP in the presence of the relevant substrate. Cells were treated with TPMP 10 μM or vehicle (deionized H_2_O), followed by 1 μM FCCP, then 1 μM AA to block the flow of electrons in the respiratory chain. **A.** Respiration on 5mM pyruvate/ 2.5 mM malate. **B.** Respiration on 5 mM glutamate / 2.5 mM malate. **C.** Respiration on 10 mM succinate. **D & E.** The acidification rate (PPR) increased following TPMP treatment in pyruvate/malate and glutamate/malate cases. **F.** In the presence of succinate, the increase in PPR occurred after AA addition. Data is expressed as the percentage of basal OCR (OCR%) and is presented as means ±95% CI, n = 4, measured in triplicate. AA, antimycin A; OCR, oxygen consumption rate; PPR, proton production rate.

In permeabilized C2C12 myoblasts, an exposure to 10 μM TPMP (approximate equivalent of 1 μM TPMP extracellular concentration in the presence of plasma membrane potential due to Nernstian accumulation) caused a rapid decrease of respiration on pyruvate/malate to 26.17% [23.21–29.12%] and of glutamate/malate respiration to 26.08% [24.66–27.50%] of basal respiration (Fig 4). When succinate was used as a substrate, the respiratory rate following a TPMP addition decreased only slightly to 89.17% [87.54–90.79%]. Adding an uncoupler (FCCP) caused only a moderate increase in respiration, which corresponds well to previous studies in permeabilized cells [37,38]. The inhibition of respiration was associated with an increase in the proton production rate (PPR, analogous to ECAR in intact cells).

### Mechanism of the inhibitory effect of TPMP—respiratory and metabolic enzymes

**Fig 5:**
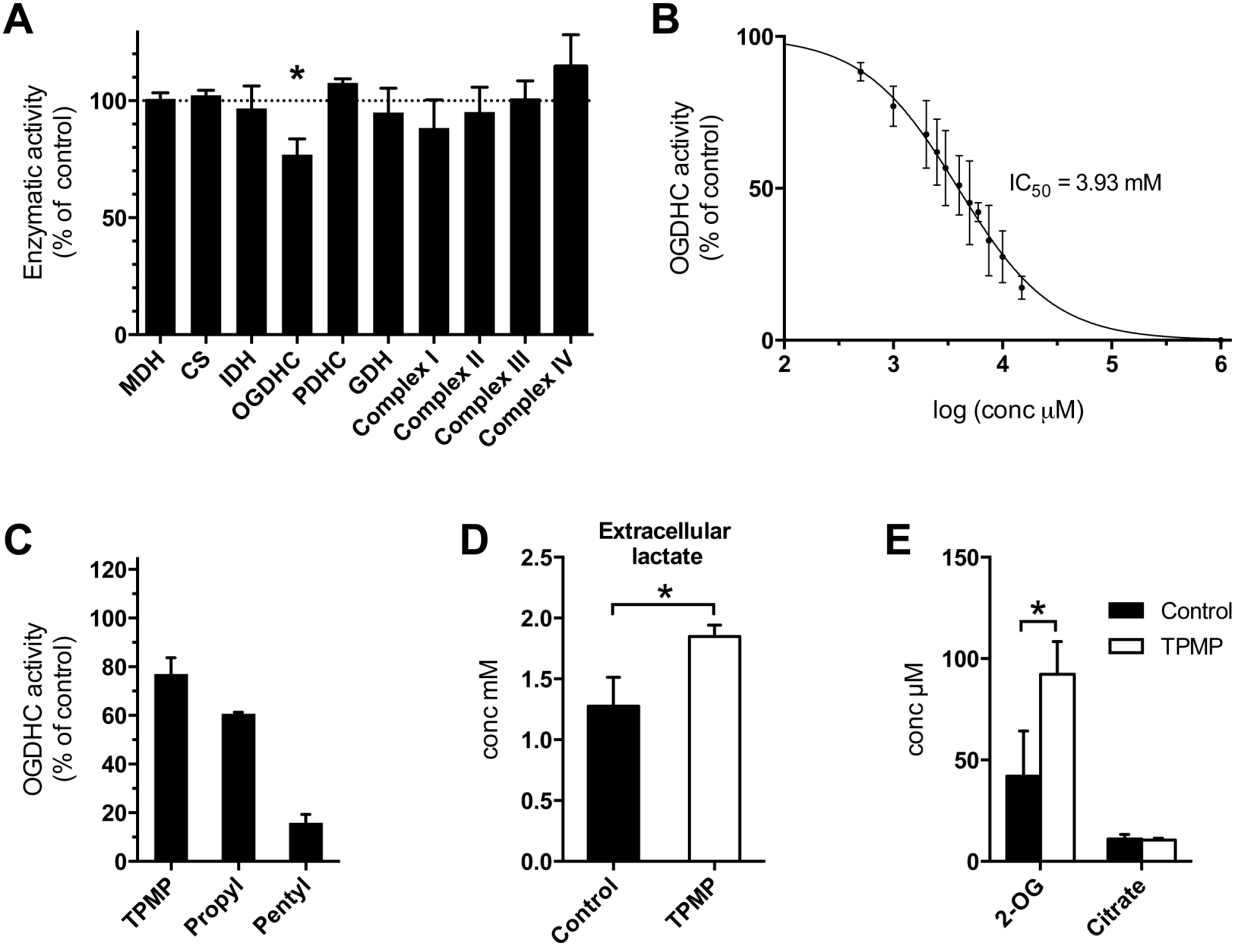
Inhibition of OGDHC by TPMP. **A.** The enzymatic activities of Krebs cycle and electron transport chain were not affected by the presence of 1 mM TPMP, except the OGDHC, which was significantly reduced. Data is presented as means ±95% CI, n = 3. * indicates significant p<0.05. Respiratory chain complexes and Krebs cycle enzymes were assessed in rat skeletal muscle homogenate enriched in mitochondrial fraction that was exposed to 3 freeze-thaw cycles, with triton X-100 0.1% included in the reaction buffer to ensure complete permeabilization of any remaining intact mitochondria. Pyruvate dehydrogenase complex was obtained purified from porcine heart. MDH, malate dehydrogenase; CS, citrate synthase; IDH, isocitrate dehydrogenase; OGDHC, 2-oxoglutarate dehydrogenase complex; PDHC, pyruvate dehydrogenase complex; GDH, glutamate dehydrogenase. **B.** IC_50_ of TPMP was found to be 3.93 [3.70–4.17] mM. Data is presented as means ±95% CI, n = 3. **C.** Longer chain alkyl-TPP^+^ compounds have higher inhibitory effect. OGDHC activity in the presence of 1 mM concentration of the more lipophilic TPP^+^ moieties in the assay mixture. The rate of inhibition was proportional to the increase in the length of the alkyl side chain. OGDHC activity was measured as in A. Data is presented as means ±95% CI, n = 3. TPMP, methyltriphenylphosphonium; Propyl, propyltriphenylphosphonium; Pentyl, pentyltriphenylphosphonium. **D. & E.** Comparison of the metabolite level in the vehicle treated control (deionized H_2_O) and TPMP treated cells in a cellular suspension (≈4 million cells in 10 ml DMEM). **D.** The concentration of lactate in the supernatant increased in the TPMP treated group after 1 hour of incubation. Data is presented as means ±95% CI, n = 4. *, p = 0.0023. **E.** The level of 2-oxoglutarate increased in the cellular pellet in the TPMP treated cells after inhibition of OGDHC complex. Data is presented as means ±95% CI, n = 4. *, p = 0.0012.

Since the previous set of experiments showed a site of inhibition upstream of complex II, we studied the activity of mitochondrial enzymes exposed to 1 mM TPMP (approximate equivalent of 1 μM extracellular TPMP, once again assuming about 1000-fold accumulation due to membrane potential). We observed negligible inhibition of respiratory chain complexes and most enzymes of the Krebs cycle, however, there was a significant inhibition of the OGDHC (Fig 5A). When treated with 1 mM TPMP, the activity of OGDHC was reduced to 77.00% [70.34–83.66%] of the untreated control. The IC_50_ of TPMP was estimated to be 3.93 [3.70–4.17] mM (Fig 5B).

### More hydrophobic TPP+ derivatives cause stronger inhibition of OGDHC

We also tested two longer alkyl chain TPP^+^ derivatives with respect to OGDHC inhibition in muscle homogenate enriched in mitochondrial fraction: 1 mM propyl-TPP+ reduced the activity to 60.70% [60.09–61.31%] of the untreated control, while pentyl-TPP^+^ caused even more inhibition to reach 15.87% [12.44–19.30%] of the control activity. Increasing the length of the alkyl side chain therefore appears to enhance the inhibitory effect on OGDHC activity (Fig 5C). Activities of pyruvate dehydrogenase complex and other Krebs cycle enzymes were not affected by the more hydrophobic TPP^+^ moieties (S2 Fig).

### Metabolite analysis

The level of extracellular lactate increased in the TPMP treated cells (Fig 5D), which supports the increase in the ECAR observed with the analysis of metabolism (Fig 1B). The analysis of intracellular metabolites showed an accumulation of 2-oxoglutarate in the TPMP treated cells further supporting the hypothesis of TPMP inhibiting OGHDC also in intact cells. There was an ≈ 2-fold increase in the level of 2-oxoglutarate (Fig 5E), while there were negligible changes in the citrate level ruling out a general toxic effect. This increase in the 2-oxoglutarate/citrate ratio is indicative of the inhibition of Krebs cycle and the shift towards anaerobic glycolysis.

### TPMP causes mixed inhibition of the OGDHC complex

**Fig 6:**
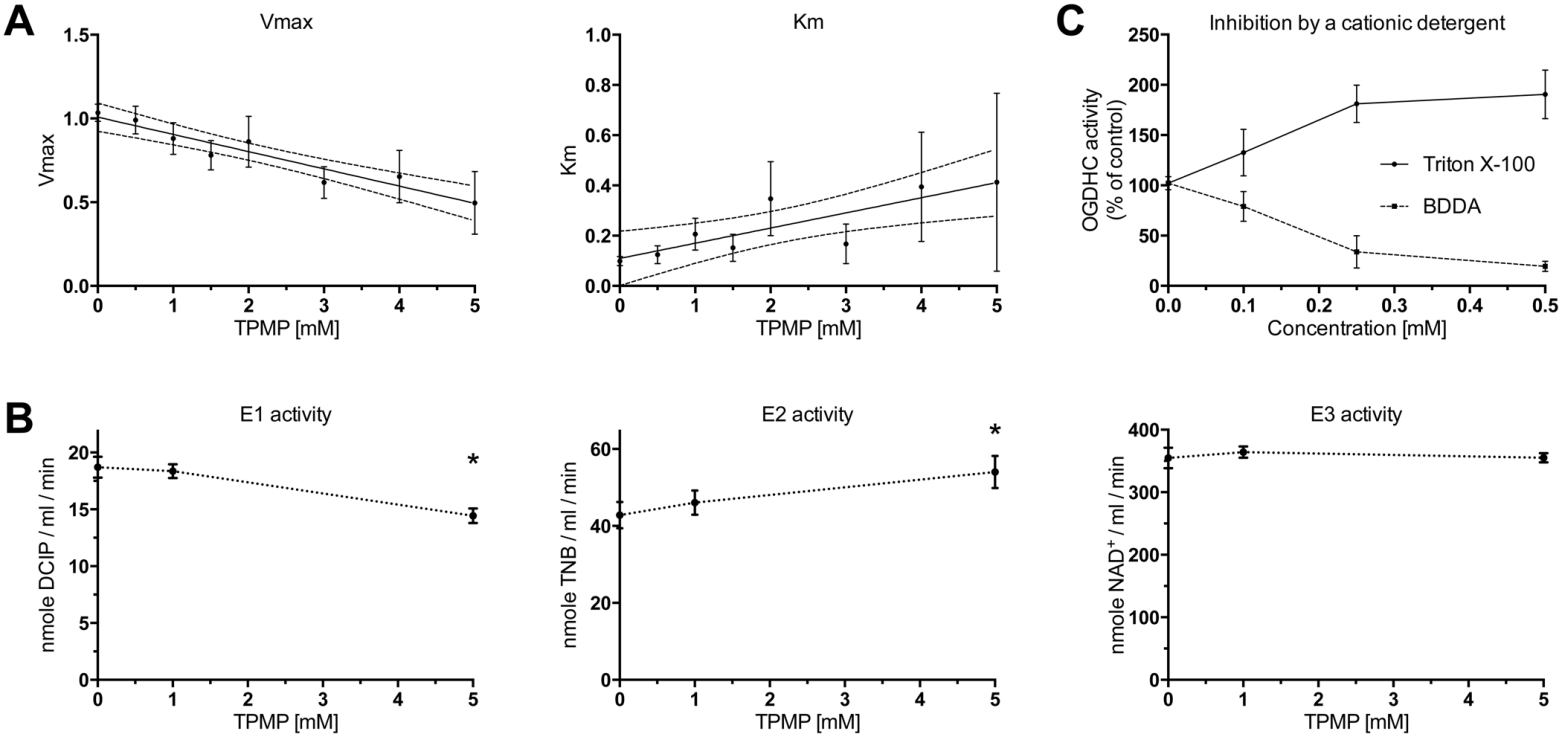
Mode and mechanism of OGDHC inhibition. **A.** TPMP causes mixed inhibition of OGDHC. Increasing the concentration of TPMP caused a decrease in the Vmax and an increase in Km. Data is presented as means ±95% CI, n = 3. **B.** E1 component of OGDHC is the most sensitive to TPMP. E2 on the contrary is activated by TPMP. E3 activity was not affected. Data is presented as means ±95% CI, n = 3. * indicates significant p<0.05. **C.** The positively charged detergent benzyldodecyldimethylammonium (BDDA) inhibits OGDHC. The non-ionic detergent Triton-X100 increases the activity. Data is presented as means ±95% CI, n = 3.

In order to determine the type of inhibition of TPMP, we plotted the values of Vmax and Km (Fig 6A) assuming the Michaelis-Menten kinetics in the presence of different TPMP concentrations. We found a high correlation between Vmax and TPMP (R^2^ 0.91), and significant correlation between Km and TPMP (R^2^ 0.69). The pattern of decreasing Vmax and increasing Km fits in a mixed-inhibition model.

### TPMP has little effect on OGDHC components

We also measured the effect of TPMP on the activity of the three components of OGDHC (Fig 6B). The activity of E1 component (alpha-ketoglutarate dehydrogenase, EC 1.2.4.2) was unaffected by 1 mM and only slightly reduced by a 5 mM concentration of TPMP. The activity of the E2 component (dihydrolipoyl transsuccinylase, EC 2.3.1.61) was actually increased in the presence of TPMP and the activity of E3 component (dihydrolipoyl dehydrogenase, EC 1.8.1.4) was not significantly affected.

### Cationic detergents exhibit similar inhibitory action as TPMP

Due to the hydrophobic nature of TPP^+^ derivatives, we were also interested whether similar detergents have similar effects. The cationic detergent benzyldodecyldimethylammonium (BDDA) exhibited a dose-dependent inhibitory action on OGDHC (Fig 6C), while the non-ionic detergent Triton X-100 caused an actual increase in OGDHC activity, as previously observed [39].

## Discussion

Triphenylphosphonium derivatives are widely used to study mitochondria and to influence its various functions including the production of reactive oxygen species [40]. The specific effects of derivatives such as MitoQ, MitoTEMPOL or MitoSOD have been well studied [5,6,8], other effects due to their chemical structure [41], however, are much less understood. Lipophilic triphenylphosphonium cations are known to interfere with mitochondrial membrane structures and respiratory chain complexes [42–44] due to their high affinity to the phospholipid membranes [45] and in this paper we show that some of the simplest and most widely used TPP^+^ derivatives also inhibit the Krebs cycle.

Our data from intact cells show a general inhibitory effect of TPMP on cellular respiration, which is considerably slower to develop compared to more lipophilic derivatives we tested previously [16]. In permeabilized cells this inhibition is much faster, probably due to an easier transport of relatively hydrophilic TPMP across the permeabilized plasma membrane [11]. Interestingly, there is a striking difference in the extent of inhibition depending on the respiratory substrate used: succinate respiration requiring only functional respiratory chain complexes II, III and IV is inhibited only to a negligible extent, while respiration on pyruvate/malate and glutamate/malate, which requires also other Krebs cycle enzymes (plus pyruvate dehydrogenase in case of pyruvate and glutamate dehydrogenase in case of glutamate) decreases substantially on exposure to TPMP. This led us to investigate further, which of these enzymes could be the site of inhibition by triphenylphosphonium derivatives.

A more detailed study of enzyme activities led to a clear candidate for the target enzyme– 2-oxoglutarate dehydrogenase complex (OGDHC), which oxidises and decarboxylates 2-oxoglutarate and attaches coenzyme A to the product to form succinyl-CoA [46,47]. We estimated the IC_50_ to be in the milimolar range, which appears high compared to other inhibitory substances, however, we must keep in mind the fact that due to the negative plasma/mitochondrial membrane potentials TPMP and its analogues can be expected to accumulate about least thousand-fold compared to their extracellular concentrations. It is therefore clear that an exposure to micromolar concentrations of TPMP and its derivatives will cause a substantial inhibition of a key Krebs cycle enzyme.

We were also interested in the type of inhibition caused by TPMP. Our kinetic analysis suggests mixed inhibition with the inhibitor binding both to the enzyme and the enzyme-substrate complex. Since the OGDHC comprises three individual subunits we were interested if TPMP affects any or all of them. Our finding that individual subunits are affected much less than the intact complex raises a question about the possible molecular mechanism through which TPMP exerts its inhibitory effect.

Alkyl TPP^+^ derivatives as lipophilic cations are similar in physicochemical properties to widely used cationic detergents (e.g. alkylammonium salts). Our observation that the cationic detergent BDDA also causes an inhibition of OGDHC while the non-ionic detergent Triton X actually increases the activity of the enzyme complex suggests a possible similarity of action between the two cationic lipophilic species.

An intriguing question arises when one compares results presented here to our previous study [16], which showed only a modest inhibitory effect of propylTPP^+^ on intact cell respiration. Why propylTPP^+^ is a more potent OGDHC inhibitor than TPMP but causes less inhibition in intact cells? One possible explanation could be that the concentration of the significantly more hydrophobic propylTPP^+^ in the mitochondrial matrix, where it is needed to interfere with OGDHC, would be lower than that of TPMP due to its preferential association with mitochondrial membranes.

Our data show for the first time that the simplest TPP^+^ derivative TPMP and its more hydrophobic analogues are inhibitors of the Krebs cycle enzyme OGDHC most likely through the disruption of the enzyme complex. This finding should be taken into account when interpreting data from experiments using TPP^+^ salts. It also opens up new research opportunities into the possibility of manipulating Krebs cycle fluxes and intramitochondrial and intracellular availability of its intermediates such as 2-oxoglutarate.

## Supporting Information

S1 Fig TPMP inhibits cellular respiration in adherent cells. The respiratory parameters concluded from measuring cellular respiration in intact C2C12 myoblasts after treatment with different concentrations of TPMP or vehicle (deionized H_2_O), followed by a sequential injection of 1 μM oligomycin, 1 μM FCCP, then 1 μM rotenone and antimycin A (R+A) mixture. ATP turnover-driven respiration is calculated by subtracting respiration after oligomycin from basal respiration (basal—oligomycin treated). Maximum respiratory capacity is calculated by subtracting the residual respiration after rotenone and antimycin A treatment from the FCCP induced respiration (uncoupled respiration—non mitochondrial respiration). H+ leak is calculated as the difference between the respiration after oligomycin addition and the residual respiration after rotenone and antimycin A treatment (oligomycin respiration—non mitochondrial respiration). Data is expressed as the percentage of basal OCR (OCR%) and is presented as means ±95% CI, n = 4, measured in triplicate.
(TIF)

S2 Fig Longer chain alkyl-TPP^+^ compounds have a higher inhibitory effect on OGDHC. The enzymatic activities of pyruvate dehydrogenase complex, glutamate dehydrogenase and other Krebs cycle enzymes were not affected by 1 mM concentration of the more lipophilic TPP^+^ moieties in the assay mixture, except the OGDHC, which was significantly reduced. Data is presented as means ±95% CI, n = 3. Respiratory chain complexes were not altered by alkyl-TPP^+^ with chains ranging from C3 to C7 [16]. PDHC, pyruvate dehydrogenase complex; CS, citrate synthase; IDH, isocitrate dehydrogenase; OGDHC, 2-oxoglutarate dehydrogenase complex; MDH, malate dehydrogenase; GDH, glutamate dehydrogenase. TPMP, methyltriphenylphosphonium; Propyl, propyltriphenylphosphonium; Pentyl, pentyltriphenylphosphonium.
(TIF)
