# Supplementary figures and images for: Mitochondrial Probe Methyltriphenylphosphonium (TPMP) Inhibits the Krebs Cycle Enzyme 2-Oxoglutarate Dehydrogenase
Source: PLoS One. 2016 Aug 18;11(8):e0161413. doi: 10.1371/journal.pone.0161413 (PMC4990249; doi:10.1371/journal.pone.0161413)

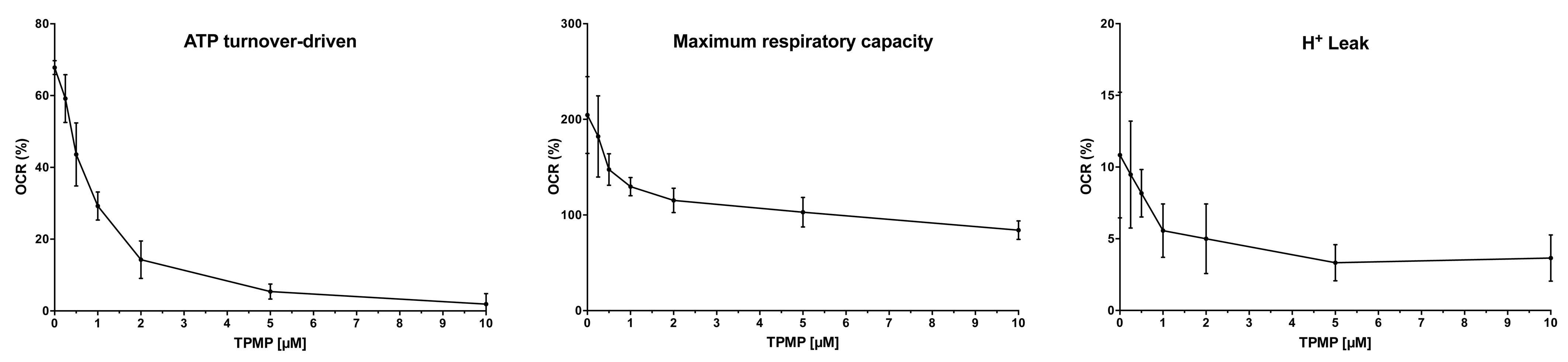

Supplement: S1 Fig — The respiratory parameters concluded from measuring cellular respiration in intact C2C12 myoblasts after treatment with different concentrations of TPMP or vehicle (deionized H2O), followed by a sequential injection of 1 μM oligomycin, 1 μM FCCP, then 1 μM rotenone and antimycin A (R+A) mixture. ATP turnover-driven respiration is calculated by subtracting respiration after oligomycin from basal respiration (basal—oligomycin treated). Maximum respiratory capacity is calculated by subtracting the residual respiration after rotenone and antimycin A treatment from the FCCP induced respiration (uncoupled respiration—non mitochondrial respiration). H+ leak is calculated as the difference between the respiration after oligomycin addition and the residual respiration after rotenone and antimycin A treatment (oligomycin respiration—non mitochondrial respiration). Data is expressed as the percentage of basal OCR (OCR%) and is presented as means ±95% CI, n = 4, measured in triplicate. (TIF) [file pone.0161413.s001.tif]

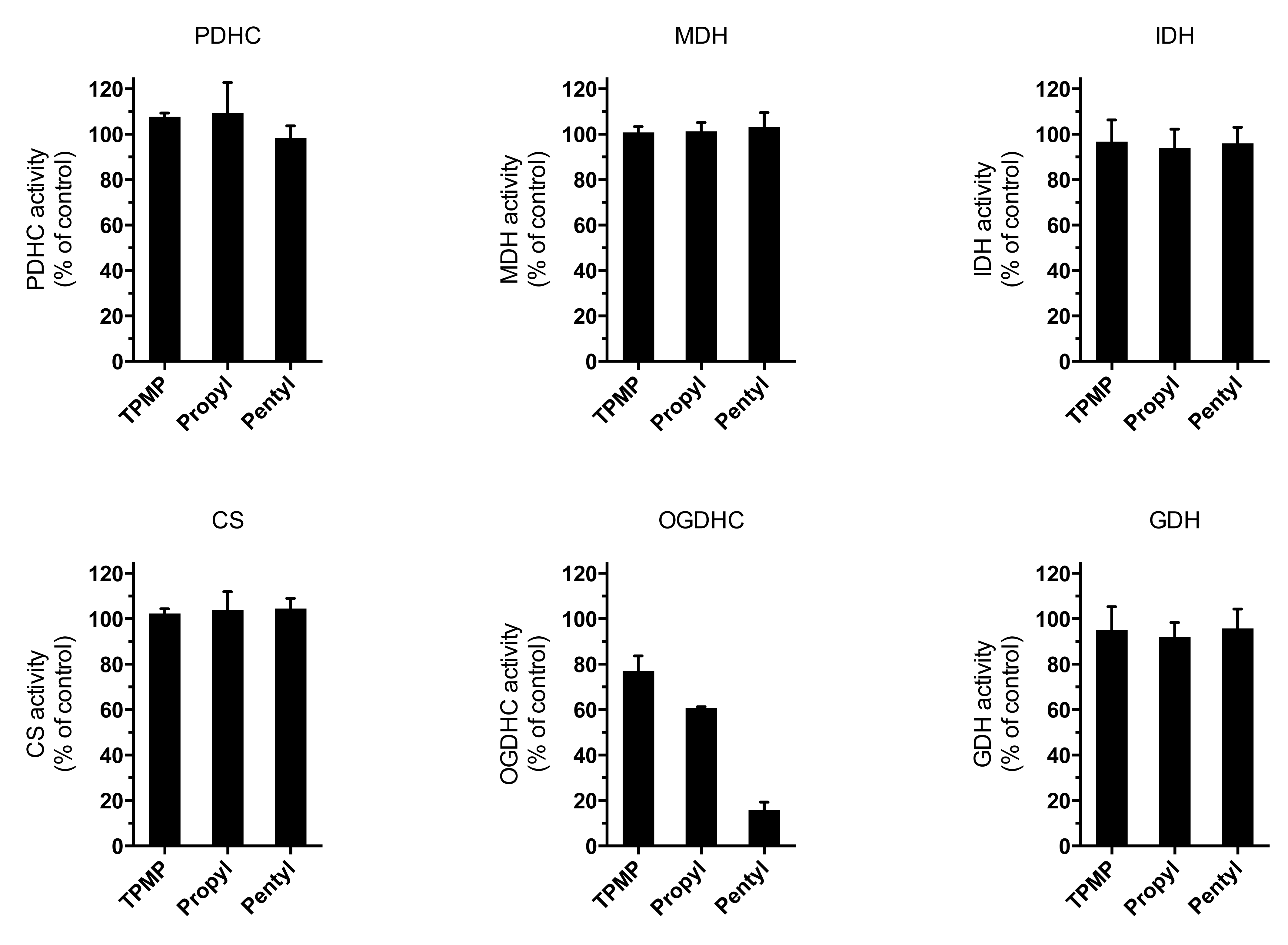

Supplement: S2 Fig — The enzymatic activities of pyruvate dehydrogenase complex, glutamate dehydrogenase and other Krebs cycle enzymes were not affected by 1 mM concentration of the more lipophilic TPP+ moieties in the assay mixture, except the OGDHC, which was significantly reduced. Data is presented as means ±95% CI, n = 3. Respiratory chain complexes were not altered by alkyl-TPP+ with chains ranging from C3 to C7 [16]. PDHC, pyruvate dehydrogenase complex; CS, citrate synthase; IDH, isocitrate dehydrogenase; OGDHC, 2-oxoglutarate dehydrogenase complex; MDH, malate dehydrogenase; GDH, glutamate dehydrogenase. TPMP, methyltriphenylphosphonium; Propyl, propyltriphenylphosphonium; Pentyl, pentyltriphenylphosphonium. (TIF) [file pone.0161413.s002.tif]
